# Pennsylvania Hospital

**Published:** 1838-01-17

**Authors:** 


### CLINICAL LECTURES.

### PENNSYLVANIA HOSPITAL.

Clinical Lectures are here delivered during November, December, January, February and March, every Wednesday morning, from 10 A. M. to 12 A. M. The physicians are Drs. B. H. Coates, Geo. B. Wood, and ———. The surgeons are Drs. T. Harris, J. Randolph, and Geo. W. Norris.

Wednesday, Dec. 27th.—At 11 o'clock, Dr. J. Randolph entered the lecture room, and commenced as follows:—We have now in the house, gentlemen, a patient with a tumour in her breast, which bears a strong resemblance to scirrhus. She has been lately attacked with inflammation in the gland, which terminated in suppuration. When the inflammatory symptoms subside, we shall then determine what course is best to pursue. I shall now avail myself of this opportunity to make some observations upon Scirrhus and Cancer.

Nothwithstanding cancer is a complaint, which has long been under the observation of surgeons, and one to which they have given their most serious attention, yet we are still imperfectly acquainted with its nature, and possess no certain means of arresting its progress. There are two forms of this affection, scirrhus, or occult cancer; ulcerated or open cancer. It may attack any portion of the system, although the glandular is most frequently its seat. It appears most generally in the female mamma; in the testicle; in the eye-ball; the uterus; the penis; the tongue; the rectum; and the lips, more especially the lower one. A true scirrhus, previous to ulceration, is a hard, dense tumor, occurring generally in a glandular part.—It often remains for a long time stationary, or advances but slowly. After a time it becomes more active; its surface is very uneven, and the tumor is usually of great weight. Severe lancinating pains accompany it, which are much complained of by the patient. The skin over the diseased portion acquires frequently a livid or leaden hue, and becomes much puckered. The superficial veins are apt to become tortuous, and varicose, and from their supposed resemblance to a crab, the ancient surgeons bestowed the name of cancer upon the disease. A genuine scirrhus always manifests a great disposition to involve the adjacent soft parts, by contracting adhesions with the skin, cellular membrane, muscles, &c. In the breast the whole gland becomes eventually involved.

A scirrhus tumour sometimes remains indolent for months, and even years; however it may advance to suppuration and ulceration in a few weeks. The substance is removed in two ways: partly by sloughing and partly by ulceration; after which a thin, acrid, sanious matter is discharged, which is capable of excoriating the adjacent parts. If you examine a genuine scirrhus tumour, when removed from the body, and after it is cut open, you will find a hard, compact central mass, from which radiate ligamentous bands, towards the circumference of the tumor. Transverse bands intersect these, forming a kind of net work, in the meshes of which, is found a softer substance. After the disease has existed for some time, it runs on to suppuration, forming an ulcer with jagged and uneven edges. Sometimes we find a central cyst filled with transparent fluid; often there is a number of these hydatids, some of them not larger than a pin's head. But from their infrequency they cannot be considered as constituting a part of the disease.—With respect to these hydatids it is thought by some writers, that they are composed of living animals. Indeed Carmichæl, Adams, and others have contended that, cancer has an independent vitality, depending upon the existence of animalcula. But microscopical observations reveal the existence of animalcula in various portions of the system. If then they are found in a healthy or sound state, why should they not also exist in diseased portions? Mr. Burns thinks that their presence afford undeniable evidence of the existence of genuine scirrhus, and is an unerring test of the propriety of an operation.

It would be fortunate if surgeons could always distinguish between tumours which present genuine scirrhus characters and such as do not. There are many tumours found in the breast, and other parts of the body, which are not scirrhus, and which yield to general and local treatment. They often arise from cold and are successfully combated by antiphlogistic means; such as bloodletting, purging, low diet, and discutients. Scrofulous tumours are often mistaken for cancerous; the female breast, and male testicle, are each subject to scrofulous diseases, bearing a strong resemblance to carcinoma. They are in general less compact and heavy, and less liable to affect the adjacent textures. Sir Everard Home acknowledges candidly that he was led into this error; that on removing tumours which he supposed to be cancerous, they on inspection proved to be scrofulous; and that tumours which he thought scrofulous turned out to be cancerous, and finally destroyed the patient's life.

When you meet with tumours of a suspicious character, the advice I give you is, first to try the usual methods for their discussion, and having continued these for a reasonable time, if they do not succeed in effecting the desired purpose, then resort to extirpation, and do not permit them, by remaining, to contaminate the system. When the neighbouring absorbent glands become diseased, it is, in general, too late to operate, for you cannot, under these circumstances, hold out to the patients or their friends any prospect of success. Sometimes however these glands become enlarged from sympathy, and upon the removal of the exciting cause, they are resolved. If you have conclusive evidence in favor of this, you may with propriety operate.

Sir Everard Home thinks that the success which is occasionally met with, in the dispersion of tumours by medical means, is rather prejudicial than otherwise; for the practitioner, deluded by his former success, perseveres in similar efforts in other cases which possess a decidedly malignant character, and protracts the period of operation until it is too late.

Another reason for not permitting tumours of a suspicious aspect to remain is this: although they may not be at first malignant, some accidental violence inflicted upon them, as blows, &c., may cause them to change their character, and to acquire malignant properties, as I mentioned to you when I lectured on tumours, the last time I had the honor of meeting you.

These remarks refer chiefly to cancer in its forming stage. When a carcinomatous tumour ulcerates a chasm is produced, as I said before, partly by sloughing and partly by ulceration. The surface of the sore is uneven; its edges are irregular, jagged and reversed; the contiguous skin is of a purple color. A discharge is poured out, which is a thin, dark colored ichor, excessively acrid, and capable of excoriating the surrounding parts. The patient suffers a tormenting, burning heat, with pain. Not unfrequently, a considerable hemorrhagy occurs from the diseased and ulcerated vessels.—Very often an attempt at reparation is made; new flesh is formed, constituting a fungus of peculiar hardness; this sometimes cicatrises, but never becomes healthy. In the mean time the disease extends itself into the system through the medium of the absorbents.

No age can be considered as exempt from scirrhus, but it most commonly occurs in advanced life. Females are believed to be particularly liable to it at the period of the cessation of menstruation.—Single women, and married women, who have never borne children, are thought to be especially subject to it. This is supposed to be owing to the breasts and uterus, not having performed the functions, for which nature designed them. It is said that this disease prevails to a great extent in the nunneries of France, attacking its victims principally between the ages of forty and fifty.

*Treatment.*—Of all the remedies, external and internal, that have been proposed for the treatment of scirrhus, no one can be relied on but extirpation. Remedies may mitigate the severity of the disorder, and perhaps arrest for a time its progress. To alleviate pain, opium and its preparations may be employed with great advantage. If inflammatory symptoms exist we may have recourse to antiphlogistics: as small bleedings, leeches, low diet, rest, fomentations, poultices, &c.

Various internal remedies have been recommended, such as hyosciamus, cicuta, digitalis, belladonna, arsenic, and the preparations of iron. These however afford but a temporary relief; in order to accomplish a cure an operation must be resorted to.

It is true that in many instances a scirrhous tumour will return, after it has been completely extirpated; but this furnishes no good argument against the operation, as it affords the only chance to the patient for life.

Two methods have been proposed for the removal of these tumours: the one by the use of the knife, the other by caustic. The knife is to be preferred. It is less painful, less tedious, and more certain in its effects. One great advantage it possesses is, that you have occular demonstration that you have removed the whole of the diseased mass. An excellent rule established in surgery, is not to be satisfied with the removal of the diseased portion only, but to include a part of the adjacent sound structure. This can be best effected by the use of the knife. The extirpation by caustic is more tedious, less certain in its results, and occasions excruciating pain. It is undeniable that escharotics have often been employed with advantage in the removal of tumours. Unfortunately, however, ignorant pretenders have seized upon them, and applied them to the treatment of this disease. The consequence has been that in many instances the knife has been postponed until too late, an incurable cancer formed, and the patient hurried to an untimely grave.

In order to impress this upon your minds, I will mention a case which occurred in this city, a few years since, strikingly illustrative of the fact.—A lady of distinguished and wealthy connections, arrived here from the South, with a small ulceration on the side of her nose. She consulted Dr. Physick who saw the ulcer, and advised its immediate removal. He offered to perform the operation himself. To this she consented. Unfortunately, however, before the time arrived for its performance, some friends persuaded her to submit herself to the treatment of an individual who had become remarkable for his treatment of scrofula and cancer. She was told of many instances of his extraordinary success, and was induced to place herself under his care. He applied some escharotic to the sore which gave her excruciating pain. It was probably the arsenious acid, or a combination of arsenic with sulphur, which you know is a very powerful escharotic. I do not know how many times he applied it, but he caused her extreme agony. A portion of the ulcer sloughed away; after which an attempt at reparation took place; granulations formed, and nearly cicatrised over for a time. The lady, I believe, thought herself cured, and the panacea gentleman, flattered himself that he had been completely successful. The affair was noised over the country, from the North to the South, and from the East to the West, and the effect perhaps was to put thousands into his pocket. I believe likewise he received a handsome fee from the lady herself. She left the city, the sore not quite healed up. Sometime afterwards she returned to Philadelphia, when I saw her in conjunction with Dr. Physick. The greater part of the side of the face was, at this time, eaten away; the bones were attacked, and she presented altogether a most hideous appearance. The termination of this appalling case of course can be well imagined.

Scirrhous disease makes its appearance as frequently in the female breast, or perhaps more so, than in any other part of the body. It appears in general as a moveable tumour, indurated and elastic, near to the nipple; unattended at first by discoloration of the skin, and remaining for some time stationary. Sooner or later, however, it enlarges; the skin now is apt to become puckered; the nipple shrinks, and is buried in the substance of the breast. The absorbent glands are enlarged and indurated. Finally the skin gives way, and an ulcer is formed, pouring out an ichorous and sanious discharge, rarely purulent.

Here is a preparation of a scirrhous mamma which I show you, exemplifying the hydatid condition of the disease, which was some years ago removed by me. This tumour was permitted to attain its present size in consequence of the practitioner who attended the patient, having been successful in some previous cases, in dispersing what he supposed to be cancerous tumours. It progressed however, until at last it obtained this great size, and the whole gland became involved in the disease.

Here is another preparation, exhibiting a scirrhous condition of the pylorus; it has somewhat lost its character, owing to long immersion in alcohol. You will observe that the line of demarcation between the healthy and diseased portions is extremely well marked. The upper portion of the duodenum is also involved, and the pancreas has taken on the diseased action. The absorbent glands in the neighborhood of the parts, are likewise in a state of disease, and the aorta itself is attached to the stomach by adhesions.

The manner of removing cancerous tumours from the breast, depends, in some measure, upon their size. If small, one incision through the integuments is sufficient. Take care to extirpate more than the diseased mass, and always divide the tumour when removed, in order to assure yourself that you have removed all that may be diseased. When large you must make two semilunar incisions, including the nipple, in the direction of the fibres of the pectoral muscle, or the long diameter of the tumour. Make your lower incision first. The reason for this is, that if the upper one be first made, you have the blood flowing over the parts, and impeding your view. Always take up all large arteries at the instant they spring; do not wait until the completion of the operation. If you do, your patient becomes faint and weak; the vessels retract and cease to bleed; you dress the wound, put her to bed, and in a few hours after reaction comes on; the vessels pour out blood, and you are annoyed by secondary hemorrhagy. To arrest this, you have to take off the dressings, open the wound, search for the vessels, and take them up. This occasions the patient more agonizing pain, and is altogether more distressing, than the primary operation.

